# Distribution of Response Time, Cortical, and Cardiac Correlates during Emotional Interference in Persons with Subclinical Psychotic Symptoms

**DOI:** 10.3389/fnbeh.2016.00172

**Published:** 2016-09-08

**Authors:** Lisa K. B. Holper, Alekandra Aleksandrowicz, Mario Müller, Vladeta Ajdacic-Gross, Helene Haker, Andreas J. Fallgatter, Florence Hagenmuller, Wolfram Kawohl, Wulf Rössler

**Affiliations:** ^1^Department of Psychiatry, Psychotherapy, and Psychosomatics, University Hospital of Psychiatry ZurichZurich, Switzerland; ^2^The Zurich Program for Sustainable Development of Mental Health Services, University Hospital of Psychiatry ZurichZurich, Switzerland; ^3^Translational Neuromodeling Unit, Institute for Biomedical Engineering, University of Zurich and ETH ZurichSwitzerland; ^4^Department of Psychiatry and Psychotherapy, University of TübingenTübingen, Germany; ^5^LEAD Graduate School, University of TübingenTübingen, Germany; ^6^Laboratory of Neuroscience (LIM27), Institute of Psychiatry, University of São PauloSão Paulo, Brazil; ^7^Department of Psychiatry and Psychotherapy, Charité University MedicineBerlin, Germany

**Keywords:** epidemiology, paranoid ideation, psychoticism, emotional Stroop, delta plot, quantile analysis, fNIRS

## Abstract

A psychosis phenotype can be observed below the threshold of clinical detection. The study aimed to investigate whether subclinical psychotic symptoms are associated with deficits in controlling emotional interference, and whether cortical brain and cardiac correlates of these deficits can be detected using functional near-infrared spectroscopy (fNIRS). A data set derived from a community sample was obtained from the Zurich Program for Sustainable Development of Mental Health Services. 174 subjects (mean age 29.67 ± 6.41, 91 females) were assigned to four groups ranging from low to high levels of subclinical psychotic symptoms (derived from the Symptom Checklist-90-R). Emotional interference was assessed using the emotional Stroop task comprising neutral, positive, and negative conditions. Statistical distributional methods based on delta plots [behavioral response time (RT) data] and quantile analysis (fNIRS data) were applied to evaluate the emotional interference effects. Results showed that both interference effects and disorder-specific (i.e., group-specific) effects could be detected, based on behavioral RTs, cortical hemodynamic signals (brain correlates), and heart rate variability (cardiac correlates). Subjects with high compared to low subclinical psychotic symptoms revealed significantly reduced amplitudes in dorsolateral prefrontal cortices (interference effect, *p* < 0.001) and middle temporal gyrus (disorder-specific group effect, *p* < 0.001), supported by behavioral and heart rate results. The present findings indicate that distributional analyses methods can support the detection of emotional interference effects in the emotional Stroop. The results suggested that subjects with high subclinical psychosis exhibit enhanced emotional interference effects. Based on these observations, subclinical psychosis may therefore prove to represent a valid extension of the clinical psychosis phenotype.

## Introduction

Several studies in the past 20 years have demonstrated that the expression of a psychosis phenotype can be observed below the threshold of its clinical detection (van Os et al., 2000; Wiles et al., 2006; Rössler et al., 2007, 2013a,b, 2015). Subclinical psychotic symptoms are commonly referred to as psychotic-(like) experiences, proneness to psychosis, at-risk mental state, schizotypy, or exceptional experiences (Fach et al., 2013). These symptoms, although not always of clinical relevance (Johns and van Os, 2001), can have predictive power for the onset of clinical psychotic disorders later in life (van Os et al., 2000). People with a high score of subclinical psychotic symptoms have been reported to have a 10% increased risk to develop a schizophrenia-spectrum disorder (Meehl, 1990; Chapman et al., 1994; Hanssen et al., 2005). As such, the topic of subclinical psychosis has gained increased interest within the context of early identification and treatment of persons at risk for psychosis.

People with subclinical psychosis indicate the same symptoms as persons with full-blown schizophrenia, though in an attenuated form. For these reasons a new diagnostic entity, labeled ‘*Attenuated Psychosis Syndrome,’* has been considered for inclusion in the new DSM-5. It is described as a condition ‘with recent onset of modest psychotic-like symptoms and clinically relevant distress and disability’ (Tsuang et al., 2013). However, because this new category did not possess any certainty of its validity, it was not yet included in the last revision of the DSM. Therefore, in order to validate subclinical psychotic states as an ‘valid’ extension of the psychosis phenotype (i.e., the typical symptoms of full-blown schizophrenia but in an attenuated form), it is necessary to demonstrate that subclinical psychosis is reflected also in other dimensions than psychopathology. In particular, it is of interest to detect underlying physiological markers that could serve as validation.

The present study aimed to answer the question of whether emotional interference effects (i.e., commonly associated with schizophrenia-spectrum disorders) can be detected in persons with subclinical psychotic symptoms. On an *emotional level*, persons with schizophrenia display deficits in the identification of information with emotional valence, together with impaired regulation of emotional behavior (Phillips et al., 2003). Typical examples of such emotional impairments have been recently reviewed (Kring and Moran, 2008), such as in the areas of reduced emotional expression (i.e., blunted or flat affect, less facially and vocally expressive responses), reduced emotional experience (i.e., anhedonia, inability to experience pleasure), and altered feelings ratings (i.e., experiencing emotions that are not necessarily associated with a given emotional valence). On a *cognitive level*, the range of cognitive impairments in individuals with schizophrenia is broad, with the more robust and replicable deficits typically found in the domains of processing speed, working memory, selective attention (Goff et al., 2011), and executive functions, such as updating, shifting, and inhibiting cognitive processes as assessed by the original Stroop task (Westerhausen et al., 2011). Impairments in these domains are associated with alterations in neural systems known to support these cognitive functions, including the prefrontal cortex (PFC) (Minzenberg et al., 2009) and the temporal lobe (Heckers, 2001; Reichenberg and Harvey, 2007). Taking these two levels together, deficits in emotion discrimination and cognitive inhibition are frequently co-occurring impairments in individuals with schizophrenia-spectrum disorders (Kohler et al., 2000).

We had the opportunity to test these issues in a data set from a community sample derived from the Epidemiology Survey of the Zurich Program for Sustainable Development of Mental Health Services (ZInEP) (Ajdacic-Gross et al., 2014). This sample was thoroughly characterized both on a psychopathological and neuropsychological level, complemented with a suitable brain imaging method, i.e., functional near-infrared spectroscopy (fNIRS) (Ehlis et al., 2014) in order to investigate cortical responses associated with potential interference deficits.

The first objective of the present study was to examine in this community sample, how emotional interference would be modified in subjects with high versus low subclinical psychotic symptoms. Subclinical psychotic symptoms were assessed using a self-rating scale based on the subscales ‘Schizophrenia Nuclear Symptoms’ (SNS) and ‘Schizotypal Signs’ (STS) (Rössler et al., 2007), derived from the Symptom Checklist-90-R (SCL-90-R) subscales ‘Paranoid Ideation’ and ‘Psychoticism’ (Derogatis, 1977).

As measure of emotional interference, we applied the color-word emotional Stroop task (Williams et al., 1996; Dresler et al., 2012). In this task the slowing of response times (RTs) for color naming of emotional words (positive and negative) relative to neutral words serves as a measure of emotional interference. There have been promising findings in patient populations, where the emotional Stroop task has been shown to be sensitive in individuals with deficits in emotional processing (Williams et al., 1996; Bar-Haim et al., 2007). Behavioral studies investigating the emotional Stroop in persons with schizophrenia have shown overall significant emotional interference effects in these patients (Phillips et al., 2005; Mohanty et al., 2008; Strauss et al., 2008; Besnier et al., 2011, 2009) (exception Demily et al., 2010). In particular, performance in persons with schizotypy (Mohanty et al., 2008) and schizophrenia (Phillips et al., 2005) differed from healthy controls by means of *increased* RTs predominantly for the emotional valence of negative words. Based on these behavioral studies, we hypothesized that subjects with high subclinical psychotic symptoms would also exhibit increased RTs to words with emotional valence; we expected this contrast to be prominent for words with emotionally negative valence.

The second objective was to examine, using fNIRS, whether there exist cortical hemodynamic and cardiac correlates of emotional interference during the emotional Stroop task. Neuroimaging studies investigating the emotional Stroop using functional magnetic resonance imaging (fMRI) in healthy subjects reported increased activity in dorsolateral, orbitofrontal, parietal, and temporal cortices in response to color processing of emotionally valence negative compared to neutral words (Compton et al., 2003). However, so far few neuroimaging studies have investigated the emotional Stroop task in people with schizophrenia-spectrum disorders; therefore, consistent findings are scarce. An fMRI study in individuals with schizotypy reported increased activity in the right and decreased activity in the left dorsolateral prefrontal cortex (DLPFC), predominantly in response to words with emotionally negative valence (Mohanty et al., 2005). An fMRI study in patients with schizophrenia reported that relative deactivations of the cingulate gyrus and the PFC were only observed in healthy controls, whereas relative activation in the DLPFC was observed only in the patient group (Park et al., 2008). We also included cardiac correlates derived from fNIRS, in order to see whether cardiovascular responses reported in previous studies in the original Stroop task (Fauvel et al., 1996; Renaud and Blondin, 1997; Boutcher and Boutcher, 2006; Satish et al., 2015), and in the emotional Stroop (Mathewson et al., 2011) could support the interpretation of the brain correlates in terms of potential interference effects.

Based on these described neuroimaging findings, a clear hypothesis could not be formulated However, we expected differences between subjects with high compared to low subclinical psychotic symptoms in the DLPFC, as these cortical areas have been consistently reported to be affected (Mohanty et al., 2005; Park et al., 2008); we expected this contrast to be prominent for emotionally valence words.

## Materials and Methods

### Subjects

Data were obtained from the Epidemiology Survey of the Zurich Program for Sustainable Development of Mental Health Services (ZInEP), a longitudinal study on prevalence rates of common mental disorders in the Canton Zurich, Switzerland. The study design has been described in detail by Ajdacic-Gross et al. (2014). All subjects gave written informed consent. The study was approved by the ethics committee of the Canton Zurich and conducted in accordance with the Declaration of Helsinki.

The subsample included in this analysis comprising the emotional Stroop task and fNIRS examination consisted of 174 subjects, who were selected from a representative survey sample of 20–41 years old adults to participate in tests at the Center for Neuro- and Sociophysiology of our department. This subsample originated from a total of 9’829 subjects, from which 193 subjects underwent neuro-physiological testing; 19 subjects were excluded from the analysis due to missing data. Subjects receiving psychiatric medications or with a diagnosis of neuro-psychiatric diseases were excluded (Ajdacic-Gross et al., 2014).

**FIGURE 1:**
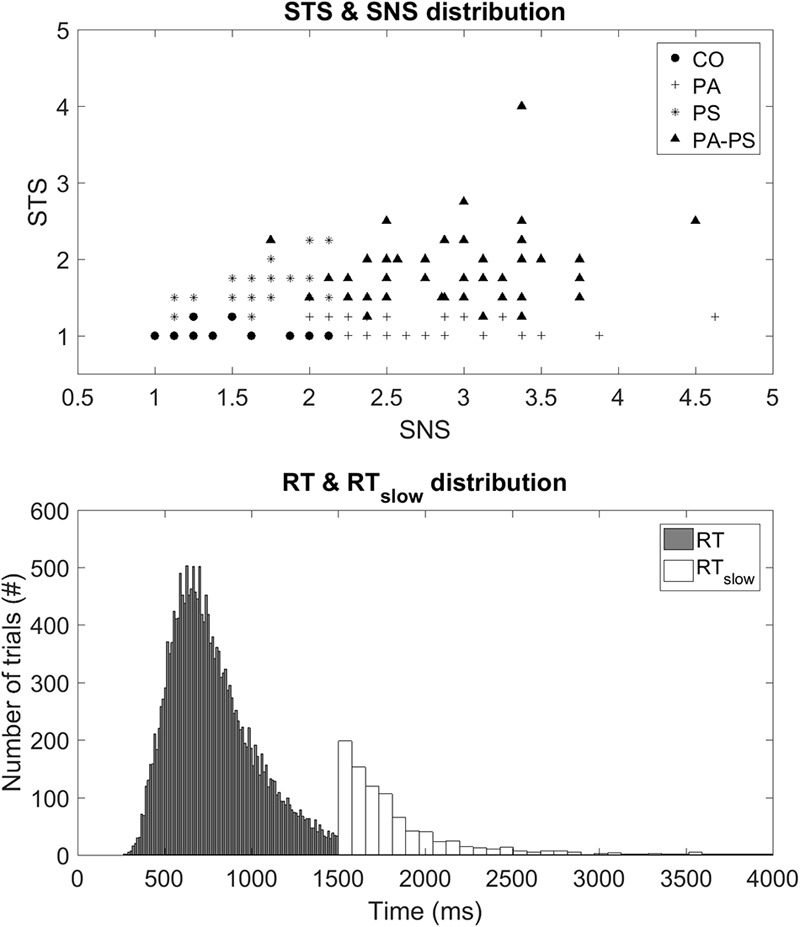
**(Top)** STS and SNS distribution. Illustration of a scatter plot demonstrating the four groups with respect to the subscores STS and SNS on the vertical and horizontal axes, respectively. **(Bottom)** RT and RT_slow_ distribution. Illustration of histogram demonstrating the distribution of the two behavioral parameters, RT and RT_slow_.

Subjects were classified based on the subscales SNS and STS (Rössler et al., 2007), derived from the Symptom Checklist-90-R (SCL-90-R) (Derogatis, 1977). The SNS subscale assesses the items, *‘Someone else can control your thoughts,’ ‘Hearing voices other people do not hear,’ ‘Other being aware of your thoughts,’ ‘Having thoughts that are not your own.’* The STS subscale assesses the items, *‘Others are to blame for your troubles,’ ‘Feeling most people cannot be trusted,’ ‘Feeling you are watched by others,’ ‘Having ideas, others do not share,’ ‘Others not giving you proper credit,’ ‘Feeling lonely even when with people,’ ‘Feeling people take advantage of you,’ ‘Never feeling close to another person.’* The SNS scale thereby subsumes four items of the original ‘Psychoticism’ scale of the SCL-90-R (thought broadcasting, hearing voices), whereas the STS scale corresponds mostly to the original ‘Paranoid Ideation’ scale. The two subscales correlated moderately positively (*r =* 0.329, *p* < 0.001). **Figure 1** illustrates the scatter plot of the four groups with the axes representing the subscores of STS and SNS, respectively.

Group CO (Control) consisted of subjects below the fifth quintiles of the two subscales (*N* = 27, 15.5%). Group PA (Paranoia) represented subjects within the fifth quintile of the STS subscale but below the fifth quintile of the SNS scale (*N* = 62, 35.6%). Group PS (Psychoticism) represented subjects within the fifth quintile of the SNS subscale but below the fifth quintile of the STS scale (*N* = 34, 19.5%). Group PA-PS (Paranoia-Psychoticism) represented subjects from both the fifth quintile of the SNS and STS subscales (*N* = 51, 29.3%). There were no significant differences between groups regarding gender, marital status, number of children, professional education, or occupation as assessed by multivariate ANOVA with the between-subject factor ‘group’ (**Table 1**).

**Table 1:** Data set.

	Demographics				
	CO	PA	PS	PA-PS	Total
Number	27	62	34	51	174
Age (mean)	29.86	30.13	29.67	29.02	29.67
Age (STD)	5.784	7.091	6.428	6.346	6.41
Male	16	19	21	27	83
Female	11	43	13	24	91
Single	19	47	22	41	129
Married	6	11	11	9	37
Divorced	2	4	1	1	8
Children yes	4	13	10	8	35
Children no	23	49	24	43	139
Obligatory school completed	27	62	34	51	174
Employed (full-time ≥ 30 h)	13	36	19	27	95
Employed (part-time < 30 h)	5	21	9	11	46
Not employed	9	5	5	12	31
	
	**SCL-90-R**				
	
	**CO**	**PA**	**PS**	**PA-PS**	**Total**
	**Mean ± STD**	**Mean ± STD**	**Mean ± STD**	**Mean ± STD**	**Mean ± STD**
	
Depression	1.426 ± 0.397	2.152 ± 0.729	1.868 ± 0.493	2.465 ± 0.814	2.073 ± 0.754
Dysthymia	1.491 ± 0.478	2.456 ± 0.654	2.280 ± 0.728	2.735 ± 0.809	2.352 ± 0.799
Somatization	1.198 ± 0.222	1.718 ± 0.510	1.701 ± 0.561	2.032 ± 0.706	1.726 ± 0.612
Anxiety	1.104 ± 0.224	1.484 ± 0.605	1.300 ± 0.369	1.824 ± 0.775	1.489 ± 0.632
Phobic anxiety	1.296 ± 0.410	2.274 ± 0.855	1.912 ± 0.642	2.750 ± 0.791	2.191 ± 0.882
Hostility	1.463 ± 0.437	2.540 ± 0.587	1.801 ± 0.447	2.980 ± 0.719	2.358 ± 0.806
Paranoid ideation	1.338 ± 0.294	2.510 ± 0.492	1.710 ± 0.296	2.883 ± 0.553	2.281 ± 0.730
Psychoticism	1.019 ± 0.067	1.089 ± 0.129	1.625 ± 0.256	1.845 ± 0.468	1.402 ± 0.455
Aggression	1.37 ± 0.271	2.278 ± 0.590	1.676 ± 0.367	2.242 ± 0.793	1.402 ± 0.685

***(Top)** Demographic data. Data were obtained from the Epidemiology Survey of the Zurich Program for Sustainable Development of Mental Health Services (ZInEP) (Ajdacic-Gross et al., 2014). There were no significant group-differences regarding age, gender, marital status, number of children, professional education, or occupation. **(Bottom)** SCL-90-R. The total sample consisting of 174 subjects was assigned to four groups (CO, PA, PS, PA-PS) based on the subscales ‘Schizophrenia Nuclear Symptoms’ (SNS) and ‘Schizotypal Signs’ (STS) (Rössler et al., 2007), derived from the Symptom Checklist-90-R (SCL-90-R) (Derogatis, 1977). Reliability testing revealed a high level of internal consistency represented by a Crohnbach’s alpha value of 0.884.*

### Emotional Stroop Task

The color-word emotional Stroop task applied in this study consisted of a total of 120 stimuli (German words) varying in their emotional valence. Forty neutral trials [‘Monat’ (month), ‘Säule’ (column), ‘Papier’ (paper), ‘Bleistift’ (pencil), ‘Zeilen’ (rows), ‘Schnur’ (cord), ‘Laterne’ (lantern), ‘Kopien’ (copies), ‘Zitate’ (citations), ‘Rohre’ (pipes)], 40 positive trials [‘Geschenk’ (present), ‘Freundlichkeit’ (cordiality), ‘Schönheit’ (beauty), ‘Umarmung’ (embrace), ‘Beifall’ (applause), ‘Urlaub’ (holiday), ‘Schatz’ (treasure), ‘Zuneigung’ (sympathy), ‘Glück’ (luck), ‘Frühling’ (spring)], and 40 negative trials [‘Alptraum’ (nightmare), ‘Geiseln’ (hostage), ‘Gestank’ (stench), ‘Infektion’ (infection), ‘Leiche’ (cadaver), ‘Scheidung’ (divorce), ‘Stress’ (stress), ‘Tod’ (death), ‘Verräter’ (betrayer), ‘Trauer’ (sorrow)]. Each word was presented in red, green, blue, and yellow on a black computer screen using Presentation software (Neurobehavioral Systems, Albany, CA, USA). The words in all three conditions did not differ regarding the number of letters and syllables, and their frequency in German language was similar. The words were presented randomly in an event-related design with an inter-trial-interval varying between 4 and 8 s. Presentation of each word took 1500 ms and was preceded by a white fixation cross shown for 500 ms. After the stimulus presentation, subjects were asked to indicate the color of the word as fast as possible by pressing a corresponding color key on a keyboard. Performance in all conditions was assessed by the response time (RT) and the error rate in response to the cues. In particular, RT was recorded during the stimulus presentation period from 250 ms until the require time of 1500 ms. If subjects did respond after the required RT of 1500 ms the trial was marked as error trial, and the time was recorded as separate parameter, in the following defined as ‘slow’ response time (RT_slow_). **Figure 1** illustrates the distribution of the two parameters, RT and RT_slow_. Trials with errors, such as no responses or corrections, were excluded.

### fNIRS Instrumentation

An ETG-4000 Optical Topography System (Hitachi, Medical Corporation, Tokyo, Japan) was used consisting of a 52-channel probe setup with 17 laser diodes and 16 photo-detectors that covered parts of prefrontal and temporal cortices (**Figure 4**). fNIRS data were recorded with a sampling frequency of 10 Hz, transformed by the modified Beer–Lambert law to concentration changes of oxy- (O_2_Hb) and deoxy- (HHb) hemoglobin. Preprocessing was done using NIRS-SPM (Ye et al., 2009), we employed the wavelet minimum description length algorithm (Jang et al., 2009) to remove systemic confounds, and the precoloring method for estimating temporal correlation (Worsley and Friston, 1995).

Motion artifacts were thoroughly removed using the following procedures. First, the Wavelet motion correction function based on the hmrMotionCorrectWavelet algorithm included in the Homer2 software (Huppert et al., 2009) was applied to all subjects. Second, data were visually inspected for remaining motion artifacts, which were subsequently removedusing NIRS-SPM (in particular, ‘steps’ and ‘spikes’) in 21 subjects.

Total hemoglobin tHb, i.e., the sum of the O_2_Hb and HHb time series, was chosen as primary hemodynamic parameter of interest because it is thought to provide higher spatial specificity for mapping cerebral activity compared to O_2_Hb or HHb separately (Grubb et al., 1974; Gagnon et al., 2012).

### Heart Rate Variability

Based on the raw fNIRS data, heart rate variability (HRV) was computed using the algorithm introduced by Scholkmann et al. (2012). The method, automatic multiscale-based peak detection (AMPD), is based on the calculation of the local heart rate maxima in the raw fNIRS time series. AMPD detects the heart rate peaks (i.e., the beats per minute), which were then used to calculate the inter-peak intervals frequency via interpolating the time difference signal. This calculation was done for each of the channels 1–52. Finally, an overall HRV estimate was obtained by computing the median heart rate frequency (Hz, beats/min) over all channels at each time point. The response variable for statistical analysis was then derived by calculating the HRV signal from each subject, trial, and condition.

### Delta Plots (Behavioral Data)

Statistical analysis was performed using Matlab (Version R2014a, The Math Works). To obtain a detailed understanding of the behavioral RT data within the emotional Stroop task, we chose a graphical technique called delta plot (De Jong et al., 1994; Ridderinkhof et al., 2005; Speckman et al., 2008; Pratte et al., 2010). Delta plots are a specific form of distributional analysis built using the quantiles of a given RT distribution. Delta plots have been proposed, by going beyond mean RT, to provide useful clues about the evaluation of the latency mechanisms underlying tasks thought to assess cognitive control and inhibition (Pratte et al., 2010; Schwarz and Miller, 2012; Unsworth et al., 2012).

**FIGURE 2:**
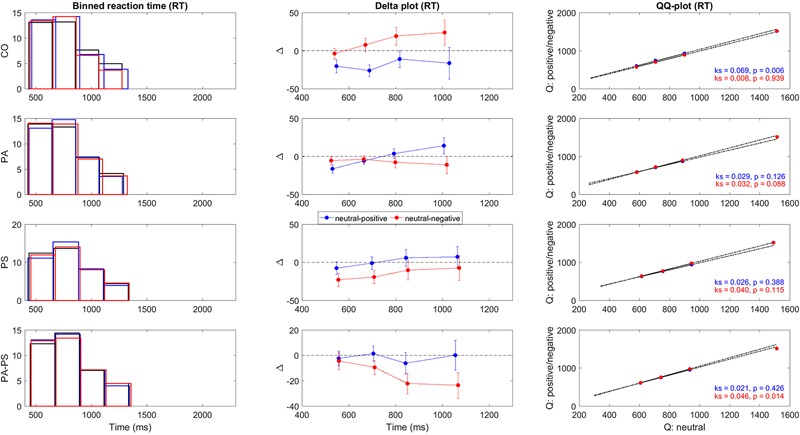
**Delta plots RT.** Illustration of the quantile differences in RT per group (CO, PA, PS, PA-PS). First panel: Binned RT frequency distributions for the neutral (black), positive (blue), and negative (red) condition. The solid lines indicate edges of the four bins. Second panel: Delta plots of quantile differences (*y*-axis, i.e., neutral minus positive, and neutral minus negative) against quantile averages (*x*-axis), as computed from the cumulative distribution functions (CDFs). Note the different *y*-units between RT and RT_slow_ (**Figure 3**), adapted for better visibility. See **Tables 3** and **4** for statistical analysis. Third panel: QQ-plots of the frequency distributions between the neutral versus the positive (blue) and negative (red) conditions. Statistical significance was assessed using two-sample Kolmogorov–Smirnov test (*k, p*-value) comparing the CDFs of the neutral versus the positive and negative conditions.

**FIGURE 3:**
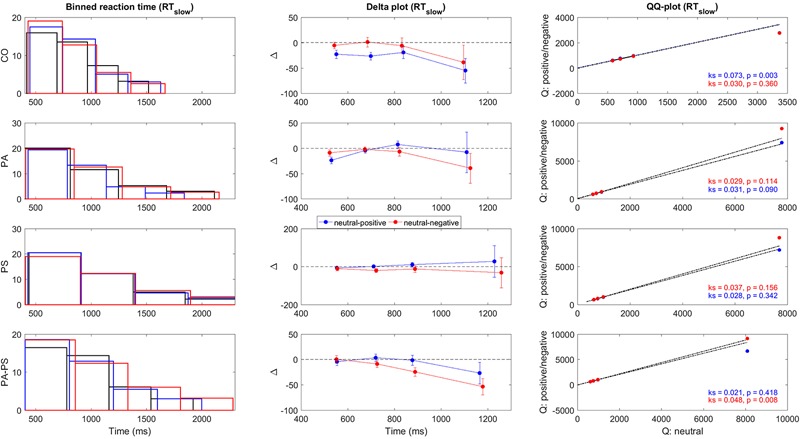
**Delta plots RT_slow_.** Illustration of the quantile differences in RT_slow_ per group (CO, PA, PS, PA-PS). First panel: Binned RT_slow_ frequency distributions for the neutral (black), positive (blue), and negative (red) condition. The solid lines indicate edges of the four bins. Second panel: Delta plots of quantile differences (*y*-axis, i.e., neutral minus positive, and neutral minus negative) against quantile averages (*x*-axis), as computed from the cumulative distribution functions (CDFs). Note the different *y*-units between RT (**Figure 2**) and RT_slow_, adapted for better visibility. See **Tables 3** and **4** for statistical analysis. Third panel: QQ-plots of the frequency distributions between the neutral versus the positive (blue) and negative (red) conditions. Statistical significance was assessed using two-sample Kolmogorov–Smirnov test (*k, p*-value) comparing the CDFs of the neutral versus the positive and negative conditions.

To construct the delta plots, we followed the procedure previously described by Schwarz and Miller (2012). The procedure was done for both RT parameters collected in this study, i.e., RT and RT_slow_. First, RTs were rank ordered for each subject and condition (neutral, positive, negative) separately and then collected to form bins of equal area. As shown in the first vertical panel of **Figures 2** and **3**, we used four bins with each bin containing 10 RTs per subject and condition (neutral, positive, negative). The group average RT within each bin was then computed and used as an estimate of the corresponding RT quantile intervals, i.e., the 0–25%, 25–50%, 50–75%, and 75–100% quantiles. Based on the RT quantiles an empirical estimate of the cumulative distribution functions (CDFs) was calculated for each condition. As shown in the second vertical panel of **Figures 2** and **3**, the delta plots were then constructed from the CDFs, by plotting the quantile differences on the *y*-axis against the quantile averages on the *x*-axis. Delta plots basically provide a visual comparison of the RT quantiles obtained in the three conditions (neutral, positive, negative). By definition, the horizontal separation of the empirical CDFs is the value of the delta plot. For example, an increase in the delta line reflects an increasing separation of two conditions (e.g., larger RTs in the neutral compared to the negative condition), whereas a drop below zero reflects a separation of two conditions in the opposite direction (e.g., larger RTs in the negative compared to the neutral condition).

To quantify the statistical significance of the quantile differences between the three conditions (i.e., *neutral minus positive*, and *neutral minus negative*), we performed two methods. First, the standard deviations of the quantile differences were calculated per condition, and statistically assessed using paired- and independent-samples *t*-test. Second, as shown in the third vertical panel of **Figures 2** and **3**, QQ-plots were obtained to serve as a visual control of the differences in quantile distributions between the three conditions (Marden, 2004), with the significances assessed using two-sample Kolmogorov–Smirnov test.

### Quantile Analysis (fNIRS Data)

For the fNIRS data, a quantile analysis was performed following the procedure described by Grinband et al. (2011) for the original Stroop task. Analog to the behavioral delta plots, the quantile analysis aimed at generating averages for each subject’s four RT quantiles. First, the tHb time series from each of the 52 channels were time-locked to the RT offset assuming a peak of the hemodynamic response at 6 s following stimulus onset (baseline corrected) (Ye et al., 2009). Likewise, HRV time series were time-locked to the RT offset assuming a peak at 3 s following stimulus onset (baseline corrected).

Second, based on the RT and RT_slow_ quantiles obtained in the behavioral analysis, Δ[tHb] beta estimates were computed using the General Linear Model (GLM) for each subject. In particular, each subject’s quantiles were computed from the RT distributions that contained all of the subject’s trials, i.e., neutral, positive, and negative, thus ensuring that the same quantiles were used for all condition types. This gave equal weight (1/4) to each quantile when comparing the Δ[tHb] beta estimates between conditions. For the HRV variable, the same quantile analysis was performed per subject.

To quantify the statistical significance of the Δ[tHb] and HRV quantile differences between the three conditions (i.e., *positive minus neutral*, and *negative minus neutral*), repeated measures ANOVA was performed using the within-subject factor ‘interference’ (neutral, positive, negative), the between-subject factor ‘group’ (CO, PA, PS, PA-PS), and the covariate ‘quantile’ (RT/RT_slow_ Q1-Q4). The Bonferroni correction was applied to counteract the problem of multiple comparisons, and results were reported at a significance level of *p* < 0.05.

## Results

To corroborate the initial group definition based on the subscales SNS and STS (Rössler et al., 2007), derived from the Symptom Checklist-90-R (SCL-90-R) (Derogatis, 1977), Fisher’s linear discriminant analysis with leave-one-out cross-validation was used. Classification results revealed that the subscales SNS and STS classified the groups to 89.5% of the cross-validated subjects correctly.

### Delta Plots (Behavioral Data)

**Table 2** illustrates an overview of the trial-based RT parameters, i.e., RT and RT_slow_. Repeated measures ANOVA revealed a main effect of ‘interference’ only for RT_slow_ (*F* = 3.142, *p* = 0.043), but not RT (*F* = 1.135, *p* = 0.321). A main effect of ‘group’ was observed for both RT (*F* = 25.876, *p* < 0.001) and RT_slow_ (*F* = 28.573, *p* < 0.001). An interaction effect of ‘interference’ × ‘group’ was observed only for RT_slow_ (*F* = 2.829, *p* = 0.009), but not RT (*F* = 0.710, *p* = 0.642). Over all groups, the largest RTs and the highest percentage of RT_slow_ were found in the negative condition (17%), followed by the positive (14%), and the neutral (13%) condition. Group CO showed a similar percentage of RT_slow_ trials (12%), compared to group PS (9%), group PA (9%), and group PA-PS (14%). However, the mean RT durations were significantly longer in groups PA and PA-PS compared to group CO. See **Table 2** for post-hoc pairwise comparisons.

**Table 2:** Response times.

		RT		RT_slow_		RT_slow_	
Group	Interference	Mean	STD	Mean	STD	Trial (#)	Trial (%)
CO	Neutral	746	276	794	308	28	3
	Positive	756	286	826	324	41	4
	Negative	713	289	809	340	57	5
	**Total**	**738**	**283**	**810**	**324**	**126**	**12**
PA	Neutral	729	270	804	430	83	3
	Positive	735	255	805	436	66	3
	Negative	737	272	819	498	81	3
	**Total**	**734**	**266**	**809**	**454**	**230**	**9**
PS	neutral	772	274	873	559	41	3
	positive	771	269	868	503	43	3
	negative	783	276	894	584	45	3
	**Total**	**775**	**273**	**878**	**549**	**129**	**9**
PA-PS	neutral	757	296	845	407	92	5
	positive	766	291	854	430	80	4
	negative	767	307	874	486	104	5
	**Total**	**763**	**298**	**858**	**441**	**276**	**14**

							***p*-Value**
**‘Interference’ pairwise comparisons**							
RT	Neutral	Positive					0.672
	Neutral	Negative					1.000
	Positive	Negative					0.672
RT_slow_	Neutral	Positive					0.828
	Neutral	Negative					0.045
	Positive	Negative					0.828
**‘Group’ pairwise comparisons**							
RT	CO	PA					1.000
	CO	PS					0.000
	CO	PA-PS					0.000
	PA	PS					0.000
	PA	PA-PS					0.000
	PS	PA-PS					0.191
RT_slow_	CO	PA					1.000
	CO	PS					0.000
	CO	PA-PS					0.000
	PA	PS					0.000
	PA	PA-PS					0.000
	PS	PA-PS					0.151

*Overview of the trial-based response time parameters, RT and RT_*slow*_. Repeated measures ANOVA revealed a main effect of ‘interference’ only for RT_*slow*_ (*F* = 3.142, *p* = 0.043), but not RT (*F* = 1.135, *p* = 0.321). A main effect of ‘group’ was observed for both RT (*F* = 25.876, *p* < 0.001) and RT_*slow*_ (*F* = 28.573, *p* < 0.001). An interaction effect of ‘interference’ × ‘group’ was observed only for RT_*slow*_ (*F* = 2.829, *p* = 0.009), but not RT (*F* = 0.710, *p* = 0.642). Shown are mean, standard deviation (STD), number (#), and percentage (%) of trials accounting for RT_*slow*_ with respect to total trials; Shown are also below the *post hoc* pairwise comparison of the ANOVA for the parameters RT and RT_*slow*_.*

Next, we constructed the delta plots. A methodological aspect should be noted. Typically, delta plots enter the faster condition first, followed by the slower condition. We chose to enter the neutral condition always first in order to obtain the delta-difference-line always by subtracting the positive and negative conditions from the neutral condition. This approach was chosen to obtain a uniform pattern in the two other conditions potentially inducing interference (i.e., positive and negative). This aspect should be considered when interpreting the present results. For example, a delta line dropping below zero, indicated a significant slowing of the corresponding (positive or negative) emotional valence conditions.

**Figures 2** and **3** illustrate the delta plots (**Figures 2** and **3**, second vertical panel) that were constructed based on the CDFs of the four bins of the RT and RT_slow_ quantiles (**Figures 2** and **3**, first vertical panel). The QQ-plots (**Figures 2** and **3**, third vertical panel) served as a statistical control comparing the CDFs between the three conditions. It should be noted that although the overlay of the bar plots in **Figures 2** and **3** may be hard to see, the overlay is an essential part of the delta plots in order to illustrate the relation to time (Schwarz and Miller, 2012).

In addition, statistical comparisons were performed for within-groups- (effect of ‘interference’) and between-groups- (effect of ‘group’) differences. The main findings regarding the effect of ‘interference’ (**Table 3**) was that groups PS (*t* = -2.619, *p* = 0.013) and PA-PS (*t* = -2.785, *p* = 0.008) responded significantly slower (larger RTs) in the negative compared to the neutral condition. This is visually illustrated by the drop of the delta line below zero. The effects were pronounced in group PA-PS (*t* = -3.301, *p* = 0.002) when considering RT_slow_. Group CO and group PA showed no ‘interference’ effects.

**Table 3:** Delta plots (effect of ‘interference’).

		CDF neutral-positive		CDF neutral-negative		CDF positive-negative	
Group	Quantile	*t*-Stats	*p*-Value	*t*-Stats	*p*-Value	*t*-Stats	*p*-Value
**RT**							
Group CO	Q1	-2.391	**0.024**	-0.563	0.578	1.666	0.108
	Q2	-3.404	**0.002**	0.860	0.398	3.921	**0.001**
	Q3	-1.010	0.322	1.614	0.119	2.816	**0.009**
	Q4	-0.781	0.442	1.442	0.161	2.196	**0.037**
Group PA	Q1	-2.976	**0.004**	-1.038	0.303	2.080	**0.042**
	Q2	-1.336	0.187	-0.752	0.455	0.411	0.683
	Q3	0.571	0.570	-1.086	0.282	-1.386	0.171
	Q4	1.325	0.190	-0.975	0.334	-2.005	**0.049**
Group PS	Q1	-0.941	0.354	-2.619	**0.013**	-1.863	0.071
	Q2	-0.145	0.885	-2.253	**0.031**	-2.016	0.052
	Q3	0.510	0.613	-0.829	0.413	-1.636	0.111
	Q4	0.522	0.605	-0.475	0.638	-1.198	0.239
Group PA-PS	Q1	-0.417	0.678	-0.659	0.513	-0.336	0.738
	Q2	0.231	0.818	-1.743	0.087	-1.636	0.108
	Q3	-0.746	0.459	-2.785	**0.008**	-2.080	**0.043**
	Q4	0.006	0.995	-2.323	**0.024**	-2.418	**0.019**
**RT_slow_**							
Group CO	Q1	-2.767	**0.010**	-0.837	0.410	1.783	0.086
	Q2	-3.550	**0.001**	0.140	0.890	3.359	**0.002**
	Q3	-1.632	0.115	-0.376	0.710	0.960	0.346
	Q4	-2.268	**0.032**	-1.143	0.263	0.491	0.628
Group PA	Q1	-3.686	**0.000**	-1.440	0.155	2.527	**0.014**
	Q2	-0.886	0.379	-0.433	0.667	0.357	0.723
	Q3	1.067	0.290	-0.799	0.428	-1.719	0.091
	Q4	-0.193	0.848	-1.334	0.187	-0.993	0.325
Group PS	Q1	-0.891	0.380	-1.083	0.287	-0.393	0.697
	Q2	0.045	0.964	-2.135	**0.040**	-2.414	**0.021**
	Q3	0.797	0.431	-0.756	0.455	-2.073	**0.046**
	Q4	0.322	0.749	-0.407	0.687	-1.135	0.265
Group PA-PS	Q1	-0.622	0.537	0.036	0.972	0.573	0.569
	Q2	0.450	0.655	-1.313	0.195	-1.691	0.097
	Q3	-0.158	0.875	-2.944	**0.005**	-2.455	**0.018**
	Q4	-1.268	0.211	-3.301	**0.002**	-1.240	0.221

*Paired-samples *t*-test assessing within-groups-differences (CO, PA, PS, PA-PS) of the cumulative distribution functions (CDFs), separately for RT and RT_*slow*_. Significant effects (*p* < 0.05) were highlighted (bold font).*

The main findings regarding the effect of ‘group’ (**Table 4**) was that groups PS (*t* = -2.166, *p* = 0.034) and PA-PS (*t* = -2.966, *p* = 0.004) responded significantly slower (larger RTs) compared to group CO (predominantly, but not only in the negative condition). The effect was preserved when considering RT_slow_. Finally, the comparison of the QQ-plots (**Figures 2** and **3**, third vertical panel) based on two-sample Kolmogorov–Smirnov test illustrated that only group PA-PS elicited a significant RT difference of the CDFs distributions between the negative compared to the neutral condition (ks = 0.046, *p* = 0.014). The effect was again pronounced when considering RT_slow_ (ks = 0.048, *p* = 0.008).

**Table 4:** Delta plots (effect of ‘group’).

		Delta neutral-positive		Delta neutral-negative		Delta positive-negative	
Group	Quantile	*t*-Stats	*p*-Value	*t*-Stats	*p*-Value	*t*-Stats	*p*-Value
**RT**							
Group PA	Q1	0.404	0.687	-0.186	0.853	-0.563	0.575
	Q2	2.465	**0.016**	-1.203	0.232	-3.247	**0.002**
	Q3	1.211	0.229	-2.023	**0.046**	-2.904	**0.005**
	Q4	1.435	0.155	-1.717	0.090	-2.901	**0.005**
Group PS	Q1	1.048	0.299	-1.666	0.101	-2.492	0.016
	Q2	2.169	**0.034**	-2.166	**0.034**	-4.083	**0.000**
	Q3	1.052	0.297	-1.676	0.099	-3.165	**0.002**
	Q4	0.973	0.334	-1.355	0.181	-2.574	**0.013**
Group PA-PS	Q1	1.752	0.084	-0.068	0.946	-1.676	0.098
	Q2	2.786	**0.007**	-1.734	0.087	-4.028	**0.000**
	Q3	0.339	0.735	-2.966	**0.004**	-3.516	**0.001**
	Q4	0.743	0.460	-2.576	**0.012**	-3.378	**0.001**
**RT_slow_**							
Group PA	Q1	-0.082	0.935	-0.339	0.736	-0.233	0.816
	Q2	2.476	**0.015**	-0.356	0.723	-2.436	**0.017**
	Q3	2.038	**0.045**	-0.044	0.965	-1.777	0.079
	Q4	0.753	0.454	-0.009	0.993	-0.903	0.369
Group PS	Q1	1.320	0.192	-0.453	0.652	-1.485	0.143
	Q2	2.387	**0.020**	-1.612	0.112	-3.976	**0.000**
	Q3	1.671	0.100	-0.300	0.766	-2.070	**0.043**
	Q4	0.852	0.397	0.066	0.947	-1.150	0.255
Group PA-PS	Q1	1.565	0.122	0.487	0.628	-0.931	0.355
	Q2	2.598	**0.011**	-0.886	0.378	-3.441	**0.001**
	Q3	1.097	0.276	-1.149	0.254	-2.220	**0.029**
	Q4	0.824	0.412	-0.449	0.655	-1.126	0.264

*Shown are *t*-test comparisons assessing between-groups-differences (PA, PS, PA-PS *compared to CO*) of the delta lines, separately for RT and RT_*slow*_. Significant effects (*p* < 0.05) were highlighted (bold font).*

### Quantile Analysis (fNIRS Data)

**FIGURE 4:**
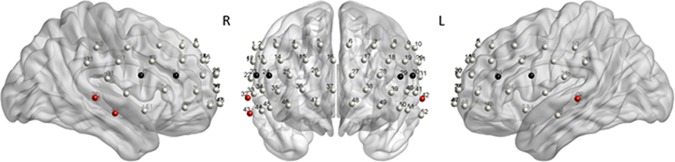
**Quantile analysis: Main effects.** Schematic front and side views of the fNIRS probe setup with 52 channels (gray). ROI DLPFC: Channels 23/24 **(right)** and 29/30 **(left)** reflected significant main effects of ‘quantile,’ ‘interference,’ and ‘group’ (black). ROI MTG: Channels 32/43 (right) and 42 **(left)** reflected a significant main effect of ‘group’ (red). The MATLAB toolbox NFRI (Singh et al., 2005) was used to estimate the Montreal Neurological Institute (MNI) coordinates of the 10–20 positions. Channels were visualized using BrainNet Viewer (Xia et al., 2013) (**Table 6**).

**FIGURE 5:**
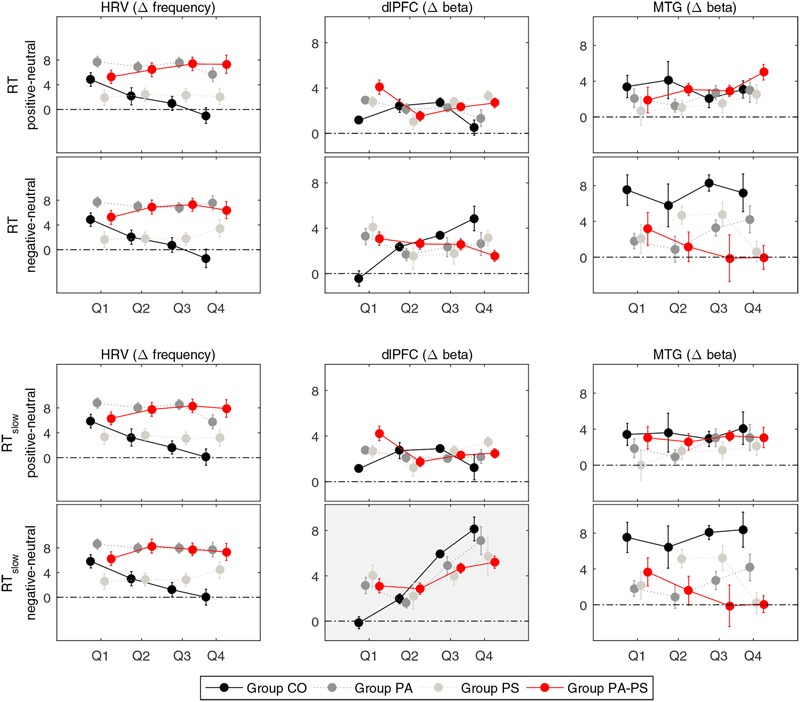
**Quantile analysis.** Illustration of the quantile differences in heart rate variability (HRV), and the Δ[tHb] beta estimates (DLPFC, MTG) per group (CO, PA, PS, PA-PS). Positive values indicate larger responses in the positive or negative compared to the neutral condition (i.e., positive minus neutral, and negative minus neutral). Error bars indicate standard error of the mean. *HRV*: Main effect of ‘group’ (*F* = 19.116, *p* < 0.001). An effect of ‘quantile’ was observed in group CO between the first and fourth quantile (Q1 vs. Q4) for RT and RT_slow_. *DLPFC*: Main effects of ‘quantile’ (*F* = 25.266, *p* < 0.001) and ‘interference’ (*F* = 25.703, *p* < 0.001) for RT_slow_. An effect of ‘group’ was observed in the fourth quantile (Q4) for RT and RT_slow_. MTG: Main effect of ‘group’ (*F* = 8.755, *p* < 0.001) and interaction effect of ‘interference’ × ‘group’ (*F* = 4.481, *p* < 0.001).

The main effects obtained by the quantile analysis are illustrated in **Figure 4**. Two regions of interest (ROIs) elicited significant effects, i.e., the DLPFC and the middle temporal gyrus (MTG). While the DLPFC [channels 23/24 (right) and 29/30 (left)] reflected effects of ‘quantile,’ ‘interference,’ and ‘group,’ the MTG [channels 32/43 (right) and 42 (left)] elicited only an effect of ‘group.’ For the repeated measures ANOVA analysis as illustrated in **Figure 5**; **Table 5**, bilateral channels of the two ROIs were collapsed over hemispheres, since no significant differences were found between hemispheres.

**Table 5:** Quantile analysis.

		RT		RT_slow_	
		*F*	*p*-Value	*F*	*p*-Value
**Main effects**					
RT vs. RT_slow_	HRV	*F* = 1.067, *p* = 0.429			
	DLPFC	*F* = 13.876, *p* < 0.001			
	MTG	*F* = 0.240, *p* = 0.624			
Quantile (covariate)	HRV	0.655	0.602	0.950	0.457
	DLPFC	0.084	0.773	25.266	0.000
	MTG	1.972	0.160	1.394	0.238
Interference	HRV	0.773	0.500	0.234	0.798
	DLPFC	0.569	0.566	25.703	0.000
	MTG	2.587	0.075	3.749	0.024
Group	HRV	20.498	0.000	19.116	0.000
	DLPFC	0.946	0.418	1.417	0.236
	MTG	8.755	0.000	8.499	0.000
Interference × Group	HRV	0.381	0.882	0.497	0.803
	DLPFC	0.362	0.903	0.235	0.965
	MTG	4.356	0.000	4.481	0.000

				**RT**	**RT_slow_**
				***p*-Value**	***p*-Value**
**‘Interference’ pairwise comparisons**					
HRV	Neutral	Positive		1.000	1.000
	Neutral	Negative		1.000	1.000
	Positive	Negative		1.000	1.000
DLPFC	Neutral	Positive		1.000	1.000
	Neutral	Negative		1.000	0.000
	Positive	Negative		0.972	0.000
MTG	Neutral	Positive		1.000	1.000
	Neutral	Negative		0.112	0.039
	Positive	Negative		0.201	0.076
**‘Group’ pairwise comparisons**					
HRV	CO	PA		0.000	0.000
	CO	PS		0.223	0.099
	CO	PA-PS		0.000	0.000
	PA	PS		0.000	0.000
	PA	PA-PS		0.031	0.148
	PS	PA-PS		0.000	0.000
DLPFC	CO	PA		1.000	1.000
	CO	PS		1.000	1.000
	CO	PA-PS		0.558	0.270
	PA	PS		1.000	1.000
	PA	PA-PS		1.000	1.000
	PS	PA-PS		1.000	1.000
MTG	CO	PA		0.045	0.011
	CO	PS		0.417	0.212
	CO	PA-PS		0.000	0.000
	PA	PS		1.000	1.000
	PA	PA-PS		0.022	0.095
	PS	PA-PS		0.009	0.026

*Repeated-measures ANOVA assessing the effects of the within-subject factor ‘interference’ (neutral, positive, negative), the between-subject factor ‘group’ (CO, PA, PS, PA-PS), and the covariate ‘quantile’ (RT/RT_*slow*_ Q1-Q4).*

**Table 6:** Regions of interest (ROIs).

ROI	Channel	MNI *x*	MNI *y*	MNI *z*	Main effects
DLPFC right	CH23	63.79	7.84	20.21	effects of ‘quintile,’ ‘interference,’ ‘group’
DLPFC right	CH24	53.62	35.84	20.05	
DLPFC left	CH29	-51.75	36.29	19.14	
DLPFC left	CH30	-61.56	9.53	19.97	
MTG right	CH32	70.76	-29.14	2.20	effect of ‘group’
MTG right	CH43	69.11	-13.07	-10.34	
MTG left	CH42	-69.13	-27.47	1.40	

*DLPFC: Channels 23/24 (right) and 29/30 (left) reflected significant main effects of ‘quantile,’ ‘interference,’ and ‘group’ (black). MTG: Channels 32/43 (right) and 42 (left) reflected a significant main effect of ‘group’ (red). The MATLAB toolbox NFRI (Singh et al., 2005) was used to estimate the Montreal Neurological Institute (MNI) coordinates of the 10–20 positions.*

Repeated measures ANOVA of the Δ[tHb] beta estimates of the DLPFC revealed a main effect of ‘interference’ (*F* = 25.703, *p* < 0.001). The effect reached significance only when considering RT_slow_, reflecting the pronouncing effect of slow RT (RT vs. RT_slow_ *F* = 13.876, *p* < 0.001). This indicated that, over all groups, larger Δ[tHb] signals were observed in the negative compared to the neutral condition. Although, no main effect of ‘group’ was observed in the DLPFC, separate pairwise comparisons revealed significant differences in the fourth quantile (Q4) for both RT and RT_slow_. This indicated that group PA-PS elicited reduced Δ[tHb] amplitudes compared to group CO in the negative condition (RT *t* = 2.304, *p* = 0.024; RT_slow_ *t* = 2.241, *p* = 0.028). Further, the DLPFC revealed a main effect of ‘quantile’ (RT_slow_ *F* = 25.266, *p* < 0.001), indicating a monotonically increasing pattern of brain activity over time.

In contrast, repeated measures ANOVA of the Δ[tHb] beta estimates in the MTG revealed a main effect of ‘group’ (RT *F* = 8.755, *p* < 0.001; RT_slow_ *F* = 8.499, *p* < 0.001), and an interaction effect of ‘interference’ × ‘group’ (RT *F* = 4.356, *p* < 0.001; RT_slow_ *F* = 4.481, *p* < 0.001). This indicated that groups PA and PA-PS elicited reduced Δ[tHb] amplitudes compared to group CO due to an interference effect during the emotionally negative valence condition. No main effect of ‘quantile’ was observed in the MTG, indicating a stable pattern of brain activity over time.

Repeated measures ANOVA of the HRV data revealed a main effect of ‘group’ (*F* = 19.116, *p* < 0.001), indicating larger HRV signals in groups PS and PA-PS, compared to group CO and PA. Although, no main effect of ‘quantile’ was observed, separate pairwise comparisons revealed significant differences between the first and the fourth quantile (Q1 vs. Q4) in group CO, both for RT and RT_slow_. This indicated that HRV signals significantly decreased over time from Q1 to Q4 in group CO in both the positive (RT *t* = 6.940, *p* < 0.001; RT_slow_ 6.822, *p* < 0.001) and the negative (RT *t* = 6. 769, *p* < 0.001; RT_slow_ 6.828, *p* < 0.001) condition. This effect was not observed in groups PA, PS, or PA-PS. Hence, the HRV reflected a monotonically decreasing pattern over time, but only in group CO (stable patterns in groups CO, PA, PA-PS).

### Comparison of Subscales

**FIGURE 6:**
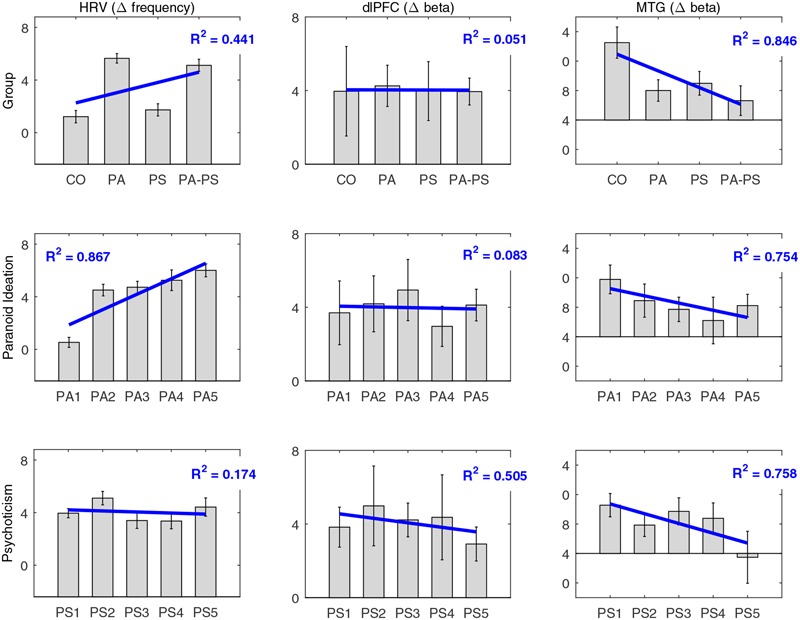
**Comparison of subscales.** Comparison of the main effects of ‘group’ (CO, PA, PS, PA-PS) with the five original quintiles of the subscales ‘Paranoid Ideation’ (PA1–PA5) and ‘Psychoticism’ (PS1–PS5), for HRV, DLPFC, and MTG. Note that for simplicity, the plots are only shown for the difference between the negative and the neutral condition based on RT_slow_. Blue lines indicate the main effect and the corresponding fit expressed as the coefficient of determination (*R*^2^).

As described in Section “Subjects,” subjects were assigned to the four groups (CO, PA, PS, PA-PS) based on the combination of the five quintiles of the two subscales STS (corresponding to SCL-90-R ‘Paranoid Ideation’) and SNS (corresponding to SCL-90-R ‘Psychoticism’). In **Figure 6**, we aimed to control whether the main effects of ‘group’ would also fit the subscales separately. The plots therefore illustrate the main effects of ‘group’ in comparison to the five original quintiles of the subscales ‘Paranoid Ideation’ (STS) and ‘Psychoticism’ (SNS). Overall, the results corresponded well to the findings in the quantile analysis. In particular, the MTG reflected a strong effect of ‘group,’ which fitted significantly with both subscales ‘Paranoid Ideation’ (PA) and ‘Psychoticism’ (PS), as indicated by a significant linear relationship for ‘group’ (*R*^2^ = 0.846), as well as for the two subscales (PA *R*^2^ = 0.754; PS *R*^2^ = 0.758). In contrast, the DLPFC showed, as expected, no effect of ‘group,’ as indicated by no linear relationship for ‘group’ (*R*^2^ = 0.051), or the two subscales (PA *R*^2^ = 0.083; PS *R*^2^= 0.505). Interestingly, the HRV data fitted significantly better with the subscale ‘Paranoid Ideation’ (PA *R*^2^ = 0.867), compared to ‘Psychoticism’ (PS *R*^2^ = 0.174; group *R*^2^ = 0.441). This may indicate that HRV as a measure of affective responsiveness reflected ‘emotional’ aspects associated with paranoid perception better, but not psychoticism. Note that for simplicity, **Figure 6** has been plotted only for the difference between the negative and the neutral condition based on RT_slow_.

## Discussion

The present work examined a community sample of individuals with low compared to high subclinical psychotic symptoms. A community sample increases the generalizability and distinguishes our work from other studies conducted in more likely biased convenience samples with subclinical psychosis (Fusar-Poli et al., 2010, 2011; Jacobson et al., 2010; Sabb et al., 2010; Modinos et al., 2011; Allen et al., 2012; Jung et al., 2012; Brent et al., 2014). The study aimed to assess deficits in the control of emotional interference in subclinical psychotic symptoms, because they have been suggested to represent a feature of schizophrenia-spectrum disorders (Kohler et al., 2000). As behavioral measure the color-word emotional Stroop task was applied. So far, no neuroimaging studies have been published evaluating the emotional Stroop in individuals with subclinical psychotic symptoms.

In the color-word emotional Stroop task (Williams et al., 1996; Dresler et al., 2012) the slowing of RTs for color naming of emotional words (positive and negative) relative to neutral words serves as a measure of emotional interference. The meaning of the emotional word stimuli is, however, semantically unrelated to the task-relevant information (ink color). Thus, it has been debated that this version of the emotional Stroop does not directly assess the interference of emotional processing with attentional processing. The lack of stable interference effects may therefore explain the contradictory findings and heterogeneity of previous results, especially in healthy controls (McKenna and Sharma, 2004; Frings et al., 2010). The primary limitation of the emotional Stroop task is that none of the previous studies could so far define a stable network related to its performance as opposed to the original color naming Stroop (Stroop, 1935) or an emotional Stroop-like task based on the semantic conflict between emotional distractor (emotional words) and emotional targets (faces) (Chechko et al., 2012). Hence, the lack of reliable behavioral effects limits the conclusions that can be drawn from previous imaging studies in healthy subjects.

For statistical quantification of the emotional interference effects, the present study applied distributional methods, i.e., delta plots and quantile analysis. These methods provided suitable frameworks to obtain a better understanding of the Stroop-dependent interference effects. The main conclusion that can be drawn from the distributional analyses is that it considers the dynamic aspect of response timing (both for the behavioral and the brain data) and that this contributed essential to the detection of interference effects and disorder-specific effects. Without considering these dynamic aspects, the present findings would have been missed. The results may therefore suggest that the contradictory findings and heterogeneity of previous studies on the emotional Stroop maybe better understood considering the present analysis.

Taking the behavioral, cortical, and cardiac level together, we could confirm all hypotheses stated in the introduction. The results therefore provided evidence that the emotional Stroop is sensitive enough to detect deficits in emotional interference in persons with subclinical psychotic symptoms, supporting an extension of the clinical psychosis phenotype.

### Delta Plots (Behavioral Data)

To obtain a detailed understanding of the behavioral RT data within the emotional Stroop task, we applied delta plots (**Figures 2** and **3**). Previous studies applied delta plots to the original Stroop paradigm (Pratte et al., 2010). Although the original Stroop paradigm and the emotional Stroop are both formally forms of interference tasks, characterized by response-relevant and response-irrelevant stimuli that can potentially produce interference effects, the two versions may nevertheless differ in the basic form of delta plots they generate (for review see Pratte et al., 2010).

The present study showed that delta plots are a suitable framework for evaluating the interference effects in the emotional Stroop task (**Figures 2** and **3**, **Tables 3** and **4**). Our main finding was that subjects with high (but not low) subclinical psychotic symptoms elicited significant interference effects in terms of a slowing of RTs in the negative condition. A significant drop of the delta line below zero visually illustrated this finding, with the largest interference effects found in the higher quantiles.

Considering the slow RTs (RT_slow_) (**Figures 2** and **3**, Bottom), the high symptom group generated slower RTs (RT_slow_) and their RTs exceeded the longest RTs generated by the CO group. This indicated that subjects with high subclinical psychotic symptoms not only showed a higher frequency of RT overshoots (>1500 ms), but also a higher magnitude of the RT maximum (exceeding the RT maximum observed in group CO). From a psychophysiological point of view, this aspect may also be associated with what may be described as ‘effect of time pressure’ (Sharma and McKenna, 2001) in the emotional Stroop task. Sharma and McKenna (2001) showed that time pressure (i.e., restricting the response interval to a required interval, as in the present study) plays an important role in determining not only the magnitude of interference effects, but also in detecting any effect at all. The authors therefore argued that time pressure may act as a stressor on its own, based on their results that interference effects of emotional valence stimuli (in particular negative stimuli) were present under high time pressure but absent under low time pressure. Hence, without (actual/perceived) time pressure no differences between neutral and emotional stimuli may be occurred. This suggests that while negative emotional stimuli do produce interference, this interference is not inevitable and may, in fact, be eliminated with longer (or un-restricted) response intervals (Sharma and McKenna, 2001). Although, the previous results may not be directly transferred to our study (since we did not assess differences between restricted versus unrestricted versions of the task), one may suggest that subjects in group PA-PS may have perceived subjective time pressure, thus leading to the observed significant interference effects.

### Quantile Analysis (fNIRS Data)

The quantile analysis allowed for a direct comparison of the dynamic aspects of the hemodynamic (Δ[tHb]) and HRV data with the behavioral delta plots. In line with previous studies (Compton et al., 2003; Mohanty et al., 2005; Park et al., 2008), brain activity in the DLPFC reflected an interference effect (**Figure 4**). Negative words stimulated significantly larger hemodynamic responses compared to neutral words. Moreover, we observed that this effect was monotonically increasing over time as a function of RT, with the largest amplitudes in the highest RT quantile. The very similar pattern in the DLPFC and the behavioral delta plots therefore suggested a strong physiological relationship of the hemodynamic with the behavioral RT data, dynamically increasing from low to high response quantiles.

From a theoretical point of view, this effect may appear similar to what has been described as time-on-task effect in the original Stroop task (Taylor et al., 2013, 2014), typically found in prefrontal areas. The time-on-task effect describes that PFC can monotonically increase neural activity with time-on-task, even when no task-related decisions are made. It has therefore been argued, that greater PFC activity on interference trials (incompatible trials in the original Stroop task) may stem from longer RTs rather than response conflict. Hence, in case of a time-on-task effect, both incompatible (high-interference) *and* compatible (low-interference) trials would show monotonically increasing activity. This argument is in contrast to the traditional conflict monitoring model (Botvinick et al., 2001), which describes that PFC is primarily involved in detecting interference between competing responses thus signaling the need for attentional control. The conflict monitoring model thereby predicts monotonically increasing neural activity with increasing RT for incompatible (high-interference) *but not* compatible (low-interference) trials. Based on these arguments, we suggest that the present results in the DLPFC do not represent a time-on-task effect, but a real interference effect based on our observation of monotonically increasing activity with increasing RT for negative (high-interference) *but not* neutral (low-interference) trials. In other words, our data suggest that DLPFC activity reflects interference effects.

In contrast to the DLPFC, the hemodynamic response in the MTG showed a stable pattern over time (**Figure 4**). The MTG rather reflected a strong disorder-specific group-effect, with reduced response in subjects with high compared to low subclinical psychotic symptoms. Generally, the temporal gyrus has been described to have gray matter volume reductions not only in patients with schizophrenia (Matsumoto et al., 2001; Onitsuka et al., 2004), but also in subclinical psychosis (Jacobson et al., 2010; Brent et al., 2014). These gray volume reductions have been reported in superior, middle, and inferior regions of the temporal gyrus, have been shown to progress over the time (Kasai et al., 2003; Takahashi et al., 2009), and to be associated with positive psychosis symptoms (Onitsuka et al., 2004). Further, fMRI studies investigating emotional priming in the original Stroop task in healthy subjects, showed a smaller response of the temporal gyrus to aversive (negative) compared to neutral priming (Hart et al., 2010). The smaller interference effect has therefore been argued to reflect functional and structural abnormalities in areas associated with language processing in the (superior) temporal gyrus in patients with schizophrenia (Li et al., 2009). The present data may be in line with this previous work, reflecting reduced magnitudes of the hemodynamic responses in temporal gyrus due to disorder-specific, but task-independent, reductions in activity. This finding might indicate that the MTG is less involved in the interference effect (compared to the DLPFC), but rather reflects disorder-specific effects.

Taking the results of the DLPFC and the MTG together, the present analysis demonstrated both interference effects and disorder-specific effects. Extending previous research, the present study thereby suggests that emotional interference in response to the emotional Stroop task can already be detected on the subclinical level of psychotic symptoms.

### Heart Rate Variability

In addition to the cortical data, the present analysis assessed individual cardiac responses. Cardiovascular responses have been reported in the original Stroop task (Fauvel et al., 1996; Renaud and Blondin, 1997; Boutcher and Boutcher, 2006; Satish et al., 2015), and in one study in the emotional Stroop (Mathewson et al., 2011). These studies consistently showed that Stroop performance was accompanied by heightened HRV levels (although also controversial results were reported, Boutcher and Boutcher, 2006). Extending previous work, we addressed disorder-specific interference effects on HRV. In particular, we show that the effects on HRV differed between groups. Subjects with low subclinical psychotic symptoms monotonically decreased in HRV levels after stimulus onset. In contrast, subjects with high subclinical psychotic symptoms maintained an increased HRV level until the end of the trial.

A potential factor that may have contributed to these findings may be the perception of stress or time pressure [see also Delta plots (behavioral data)] based on differences in subjective anxiety. For example, a previous study reported that state-anxiety scores increased during original Stroop performance, but only among subjects who completed a large number of trials with restricted response intervals, i.e., performance likely to induce stress or time pressure (Renaud and Blondin, 1997). This and other studies showed that subjective anxiety may influence performance in the emotional Stroop task. In particular, it has been argued that inconsistent findings regarding the emotional Stroop effect in healthy subjects may be explained not only by effects of stimulus valence and arousal, but also by confounding individual differences in state anxiety (not trait anxiety) (Dresler et al., 2009). This state anxiety-related effect has even been suggested in healthy subjects, indicating that arousal produces emotional interference independent of word valence, and that state anxiety exacerbates interference of emotional words by further biasing attention toward emotionally salient stimuli (Dresler et al., 2009). Taking together, it may be argued that differences in subjective perception of stress, anxiety, or time pressure played a role in the increasing HRV response observed on the present study.

## Conclusion

The present findings are the first that provide evidence of cortical hemodynamic correlates of emotional interference in subjects with subclinical psychotic symptoms using fNIRS. The results based on distributional analysis suggest that beyond the evaluation of averaged RTs, the evaluation of the dynamic aspects of response timing may support the detection of both interference effects and disorder-specific effects. Taking the behavioral, cortical, and cardiac level together, the presented study proposes that further careful investigations of prefrontal and temporal cortices may provide neurobiological correlates of subclinical psychotic symptoms during emotional Stroop performance.

The specific value of the present work is that the observed brain correlates of subclinical psychotic symptoms were obtained in a community sample. This sample presented symptoms similar to persons with schizophrenia, though in an attenuated form. It may consequently be suggested that these individuals represent what has been earlier proposed as a new DSM-5 diagnostic entity (‘Attenuated Psychosis Syndrome’) (Tsuang et al., 2013). Although the validity of our observations needs to be confirmed, the brain correlates of subclinical psychotic symptoms reported in the present work may indicate a step toward possible markers of such attenuated psychotic symptoms.

## Author Contributions

Authors WR, WK, HH, VA-G, and AF, designed the study and wrote the protocol according to the Epidemiology Survey of the Zurich Program for Sustainable Development of Mental Health Services (ZInEP). Authors FH, AA, and MM, collected the data. Author LH undertook the statistical analysis and wrote the first draft of the manuscript. All authors critically revised and approved the final manuscript.

## Conflict of Interest Statement

The authors declare that the research was conducted in the absence of any commercial or financial relationships that could be construed as a potential conflict of interest.

The reviewer VP and handling Editor declared their shared affiliation, and the handling Editor states that the process nevertheless met the standards of a fair and objective review.
